# Fire-severity effects on plant–fungal interactions after a novel tundra wildfire disturbance: implications for arctic shrub and tree migration

**DOI:** 10.1186/s12898-016-0075-y

**Published:** 2016-05-11

**Authors:** Rebecca E. Hewitt, Teresa N. Hollingsworth, F. Stuart Chapin III, D. Lee Taylor

**Affiliations:** Institute of Arctic Biology, University of Alaska Fairbanks, Fairbanks, AK 99775 USA; Center for Ecosystem Science and Society, Northern Arizona University, PO Box 5620, Flagstaff, AZ 86011 USA; US Forest Service, Pacific Northwest Research Station, Boreal Ecology Cooperative Research Unit, Fairbanks, AK 99775 USA; Department of Biology, University of New Mexico, Albuquerque, NM 87131 USA

**Keywords:** *Alnus viridis*, Arctic tundra, ARISA, Climate change, Fire severity, Fungal internal transcribed spacer (ITS), *Picea mariana*, Shrub expansion, Treeline

## Abstract

**Background:**

Vegetation change in high latitude tundra ecosystems is expected to accelerate due to increased wildfire activity. High-severity fires increase the availability of mineral soil seedbeds, which facilitates recruitment, yet fire also alters soil microbial composition, which could significantly impact seedling establishment.

**Results:**

We investigated the effects of fire severity on soil biota and associated effects on plant performance for two plant species predicted to expand into Arctic tundra. We inoculated seedlings in a growth chamber experiment with soils collected from the largest tundra fire recorded in the Arctic and used molecular tools to characterize root-associated fungal communities. Seedling biomass was significantly related to the composition of fungal inoculum. Biomass decreased as fire severity increased and the proportion of pathogenic fungi increased.

**Conclusions:**

Our results suggest that effects of fire severity on soil biota reduces seedling performance and thus we hypothesize that in certain ecological contexts fire-severity effects on plant–fungal interactions may dampen the expected increases in tree and shrub establishment after tundra fire.

**Electronic supplementary material:**

The online version of this article (doi:10.1186/s12898-016-0075-y) contains supplementary material, which is available to authorized users.

## Background

In the last half century, warming in the Arctic and Subarctic has been correlated with the expansion of tundra shrubs into graminoid tundra [1] and the migration of forest into tundra in some locations [2]. These changes in vegetation could have strong positive feedbacks to the climate system, accentuating warming, through decreases in albedo, carbon storage, and increases in landscape flammability [3, 4]. Evidence suggests that factors influencing seedling establishment are the most critical determinants of global treeline [5] and shrubline advances [6]. However, the suite of ecological factors that influence seedling establishment in novel environments beyond current range limits are still not well understood.

Soil biota may influence the capacity of boreal trees to migrate into tundra and tundra shrubs to expand into non-shrubby tundra. Vegetation establishment can be influenced by soil biota, both mutualists and pathogens, which can affect both individual performance and plant species interactions. Although microbial symbionts can strongly influence plant performance and community structure [7], their impact on landscape-scale vegetation change is often overlooked. For example ectomycorrhizal (EM) fungi, are essential to seedling establishment and growth both inside [8] and outside [9] the native range of the host plant. Compared with non-mycorrhizal seedlings, EM-seedlings may display greater nutrient acquisition, lower levels of disease, and lower drought stress [10–12]. Although less well-studied, dark septate endophytes (DSE), may also influence seedling establishment due to their suggested mutualistic influences, which largely overlap with those of EM fungi [13–15]. On the other hand, the establishment of seedlings can also be limited by the presence of species-specific enemies, including fungal pathogens [16–18]. The net outcome of negative interactions with pathogens and positive interactions with mutualists can influence the relative abundance and migration capacity of a plant species [11, 18].

The fire regime directly affects seedling recruitment success and migration [19] in the boreal forest, shrub growth and reproduction in the Subarctic [20], and shrub expansion in the Arctic [21] primarily through the effects of the severity of fire on the availability of high-quality, mineral soil seedbeds and time since fire on successional dynamics. Although fire disturbance has been relatively rare in the Arctic tundra for the last 11,000 years [22], in the last half-century the extent of tundra fires has increased due to warm and dry weather [23]. Increased fire frequency and severity in Arctic tundra is therefore expected to facilitate both tree migration and shrub expansion.

Despite the expected acceleration of tree migration and shrub expansions associated with warming in the Arctic [24], the effects of fire disturbance on fungal mutualists and pathogens may exert positive and negative indirect effects. For example, the same severe burns that increase the availability of high-quality establishment sites on mineral soil [19] can also alter the community structure of soil-dwelling fungal symbionts [25] and thus the fungal taxon-specific provision of soil resources to host plants. Recent research describes the broad geographic patterns of fungi across Arctic Alaska [26–28], yet the response of these fungal communities to wildfire disturbance is unknown. In temperate and boreal regions, severe fires decrease EM richness [29] and root colonization [30], and post-fire EM colonization of seedlings can be dependent on spores, sclerotia, or other components of the post-fire resistant propagule community (RPC) [31] as an inoculum source. Whether fire-severity affects DSE abundance and colonization in a similar manner to EM is not known. In addition to fire-effects on potentially beneficial mycobionts, burns can induce infection in fire-damaged roots of vegetation that survives fire [32], thus affecting the prevalence of pathogens that may associate with establishing seedlings. In tundra where fire has been relatively rare, the effect of fire severity on the prevalence of pathogens and availability of beneficial mycobionts in tundra is largely unknown.

Due to the historic rarity of tundra fires, current predictions of vegetation change after fire in Arctic Alaska are based on assumptions derived from the richer body of boreal forest research, which suggest that seedbed quality, i.e. exposure of moist, mineral seedbeds, is the primary ecological filter that drives seedling establishment [33]. The importance of post-fire, mineral seedbeds to seedling establishment in the boreal forest implies that in Arctic Alaska higher severity fires will produce a better seedbed and thus facilitate treeline advance and shrub expansion. As a first step towards understanding the potentially important biotic effects of root-associated fungi on tree and shrub establishment in tundra, we used a growth chamber experiment to investigate the role of post-fire soil microbes on seedling performance for two plant species predicted to migrate into tundra under future scenarios of warming and fire. In a companion field study, we observed that fire severity influenced the fungal communities associated with the dominant tundra shrub *Betula nana* and that these shrubs appear to provide a source of inoculum resilience by maintaining some mycorrhizal fungi after wildfire [34]. However, whether there is adequate inoculum in soils immediately after fire and how the inoculum varies with fire severity is unknown in tundra ecosystems. Specifically, we tested the hypothesis that increasing fire severity decreases performance in establishing seedlings due to fire-severity effects on root-associated fungal mutualists and pathogens. We examined the relationships between seedling biomass, fungal community composition, and fire severity. To our knowledge this study is the first investigation of fire-severity effects on plant–fungal interactions after tundra fire and thus provides an opportunity for hypothesis development regarding the importance of post-fire soil biota to seedling establishment, a process key to vegetation change in treeline and tundra ecosystems.

## Methods

### Study species and field sampling

In Alaska, extensive woody expansion into tundra has been documented for alder shrubs, *Alnus viridis* (Chaix) DC. [1], and its growth and reproduction are greater in burned sites [20]. Black spruce, *Picea mariana* (Mill.) Britton, Sterns & Poggenb., is the dominant latitudinal treeline species throughout most of North America. In boreal Alaska, occurrence of black spruce on the landscape is in large part determined by fire history [35] and, in addition, is often found in the coldest, wettest areas of the boreal forest. These black spruce communities share many plant species with the Alaska Arctic moist acidic tundra communities [36]. Both alder and spruce species are obligately EM [37]. Seeds of both species were collected in Interior Alaska at Washington Creek and Fairbanks and stored at −20 °C until used in our growth chamber experiment.

Between July and October 2007 the Anaktuvuk River fire (ARF), the largest tundra fire ever recorded on the North Slope of Alaska, burned 1039 km^2^ of upland shrubby tussock tundra underlain by continuous permafrost [38] approximately 100 km north of present-day latitudinal treeline. The dominant vegetation before the fire was moist acidic tundra (54 %) with moist nonacidic tundra (15 %) and shrubland (30 %) covering smaller areas [39]. We focused our study on moist acidic tundra that is dominated by sedges (*Eriophorum vaginatum* L., *Carex bigelowii* Torr. Ex Schwein), evergreen shrubs (*Ledum palustre* L. and *Vaccinium vitis*-*idea* L.) that associate with ericoid mycorrhizal fungi (ERM), deciduous shrubs (*B. nana* L. and *Salix pulchra* Cham.) that are in symbiosis with EM fungi, mosses, and lichens [40] and thus are very similar to the understory composition of some boreal black spruce forests [36]. There is overlap in the composition of EM fungi that associate with EM tundra shrubs in this region and EM host trees and shrubs in the boreal forest [27, 41, 42].

**Fig. 1:**
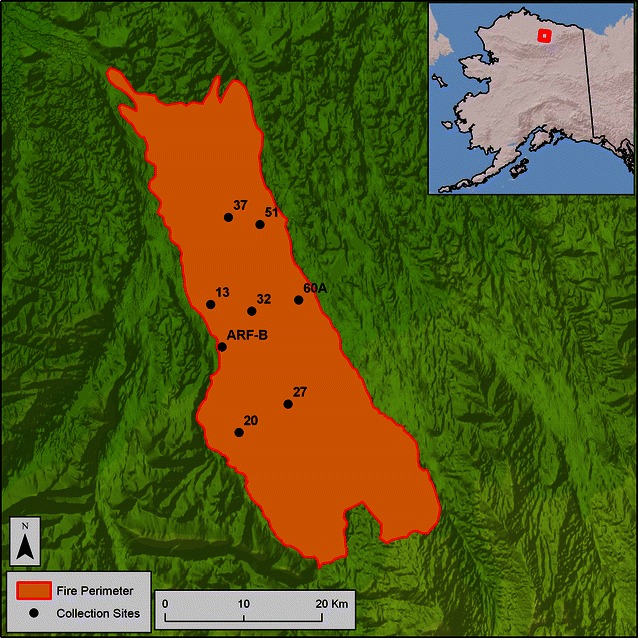
Map of soil collection sites that represent a fire-severity gradient within the Anaktuvuk river fire burn scar: low severity = sites *27* and *51*; moderate severity = sites *20*, *32*, and ARF-B; high severity = sites *13*, *37*, and *60A*

In July 2008, the first growing season after the fire, we visited eight burned sites within the ARF burn scar corresponding with different fire severities via a one-time opportunity to access the burn scar with helicopter-based logistical support and approval from the Bureau of Land Management (Fig. 1; Additional file 1). Fire severity was measured in the field as the composite burn index (CBI) and with site descriptions of post-fire vegetation composition and structure and combustion of vegetation and soil [39]. At 20 points along a 50 m transect, we collected approximately 15 ml of organic soil and 15 ml mineral soil from the top 5 cm of the soil horizon. We then pooled and homogenized our 20 samples per site by soil horizon, and stored them at 4 °C at the University of Alaska Fairbanks for 3 weeks until we inoculated the host plants.

### Experimental design

We conducted a growth chamber experiment using a randomized block design with 18 treatments and 10 replicate blocks and two host plant species. There were 16 treatments with inoculum from field soil [8 sites × 2 soil horizons (organic and mineral)] and two additional treatments to test for unintentional inoculation of seedlings in the growth chamber with sterile inoculum (twice-autoclaved mineral and autoclaved organic soils). In July 2008 we surface-sterilized black spruce seeds with a solution of 5 % household bleach, 5 % ethanol, and liquinox soap for 5 min and the smaller alder seeds for 1 min followed by ten rinses with ultrapure water. Seeds were placed in sterile petri dishes on autoclaved filter paper, and RO water was used to keep the seeds moist.

We transplanted seedlings from petri dishes into standard 150 ml cone-tainers (Stuewe and Sons, Inc., Tangent, Oregon, USA) filled with sterilized silt soil 2 weeks after germination and then inoculated them with 1 of the 18 treatments. Twelve milli-liters of treatment soil was added to the top of each cone-tainer and watered into the autoclaved substrate. Seedlings received one 5 ml application (14 ppm N) of a 9:20:9 NPK fertilizer solution one and half months after inoculation and one 5 ml application (50 ppm N) 4 months after inoculation due to the chlorotic appearance of the seedlings. Seedlings were grown in a controlled-environment chamber (Conviron CMP 3246, Winnipeg, Manitoba, Canada) for 7 months at 25/10 °C day/night with 16-h photoperiod at 300 μmol m^−2^ s^−1^ irradiance at ambient humidity (26–96 % RH, mean 73.75 RH ± 0.16 SE, NOAA National Climate Data Center http://www.ncdc.noaa.gov/). We watered seedlings to excess with RO water as needed. Height and survival were measured monthly. At the time of harvest, roots were rinsed gently with RO water and separated from shoots. We dried shoots and roots after root tip sampling (described below) at 60 °C for 48 h in a drying oven (VWR Scientific Products Forced Air Oven, Radnor, Pennsylvania, USA) and determined the dry weight of roots, stems, and leaves.

### Characterization of fungal communities

Harvested root systems were cut into 4 cm segments and floated in ultrapure water in a petri dish. Using a dissecting microscope (40× magnification) we randomly selected ten live root tips that exhibited signs of fungal colonization, e.g., no root hairs, from each root system. For each seedling, root tips were pooled for automated ribosomal intergenic spacer analysis (ARISA) and DNA sequence analysis of root-associated fungal community structure. ARISA community profiles provide information on the number and relative abundance of taxa, ribotypes, within a sample; however, ARISA does not provide taxonomic identities of the taxa. Therefore, we used Sanger sequencing to assign taxonomic identities to the dominant ARISA ribotypes (see Additional file 2). Pooled root tip samples were placed in a single 0.6 ml Eppendorf tube, frozen in a small amount of ultrapure water, and stored at −80 °C.

In September 2010 we extracted DNA from lyophilized pooled root tip samples of each seedling. From these pooled genomic DNA samples, the fungal ITS gene region was amplified using the primers ITSF and ITS4 following the protocol of Bent and Taylor [43]. We obtained ARISA fungal community profiles and ITS sequences following Bent et al. [44]. Fungal taxa were inferred from ITS sequences and matched to dominant ARISA ribotypes based on sequence and ribotype fragment lengths ([45]; Additional file 3). Fungal identities were assigned though comparison of our ITS sequences to those from GenBank using a curated fungal-ITS BLAST search (http://www.borealfungi.uaf.edu/) that excludes environmental and uncultured sequences ([46]; Additional file 2). In addition, we constructed maximum likelihood trees to infer identities for sequences with inconsistent identities resulting from the BLAST search (Additional file 2). Nomenclature for our sequences follows Timling et al. [27]. Functional groups were assigned based on Operational Taxonomic Unit (OTU) identities. For example, for species and genera we could categorize OTUs as pathogens, endophytes, DSE, saprotrophs, or ERM. OTUs that were assigned identities at family or higher levels of taxonomic resolution were assigned multiple functional groups (Additional file 4). In a few cases the closest sequence identities matched with a ribotype were from different functional groups, in which case we described ribotypes as either pathogenic or nonpathogenic (i.e. ERM, DSE).

### Statistical analysis

We used nonmetric multidimensional scaling (NMDS) [47] ordinations to interpret the variability in fungal composition across seedlings. Ordinations were based on ribotype abundance data, and we used Beal’s smoothing to relieve the “zero truncation problem” [48]. We observed some fungal colonization of roots when we ran ARISA on seedlings that received the sterile inoculum (23 ribotypes on 31 of the 40 seedlings that received the sterile treatment; five of the ribotypes occurred more than once). We examined the relative abundance of the five ribotypes that occurred more than once and found that their abundance did not vary significantly between treatments. Therefore, we made the assumption that any variability in biomass detected is due to treatment effect and not the effects of the five ribotypes. In order to adjust for this contamination we used the most conservative approach, and all ribotypes observed on these seedlings were excluded from multivariate analysis of fungal composition. Following McCune & Grace [49] we eliminated all rare ribotypes, i.e. those that occurred in less than 5 % of the samples. We used the Sorensen distance measure and a random starting configuration with a final solution generated using 500 iterations [49]. First, to determine whether fungal composition was related to seedling biomass, we ordinated the fungal communities associated with all seedlings regardless of treatment or host species. Secondly, to investigate whether treatment differences in fungal composition were related to seedling biomass, we pooled treatment replicates into one fungal community profile per treatment (i.e. combined soil horizon and burn severity) for each host plant. In both cases, axis scores were produced and then used in regression analysis as a measure of fungal composition. We also used multiple-response permutation procedures (MRPP) [49] with the Euclidean distance measure [49] to investigate whether there were differences between fungal communities in burned sites grouped by low, moderate, and high burn-severity categories (low = 2 treatments, moderate = 3 treatments, high = 3 treatments) and between soil horizons (organic = 8 treatments, mineral = 8 treatments). Fire-severity categories were defined by CBI and site severity descriptions.

We assessed normality graphically for all response variables (total biomass, shoot biomass, root biomass, root:shoot, stem weight, leaf weight, and life span,) and considered skewness, kurtosis, and Shapiro–Wilk’s W values before log-transforming data. We evaluated correlations between response variables using Spearman correlations. All response variables were significantly correlated to total seedling biomass (all correlations P < 0.05 and Spearman’s ρ > 0.14), so we used log-transformed total biomass as the response variable for subsequent analyses. We used ANOVA to investigate inoculation effects on log-transformed seedling biomass with host plant species and inoculation status as factors, and ANCOVA to investigate the relationship between log-transformed seedling biomass and treatment, which includes a continuous fire severity factor and a categorical soil horizon factor for each host species. To evaluate the relationship between fungal composition (NMDS axis scores) and log-transformed seedling biomass, we used stepwise regression to evaluate the best-fit model comprised of all or a subset of NMDS axes. We used regression to test for relationships between the proportion of functional groups of fungi and both fire severity and log-transformed seedling biomass. Stepwise regression was then used to determine whether the relative abundance of particular taxa within defined functional groups were related to log-transformed seedling biomass. We used T test to compare the survivorship between host plants. Survivorship was expressed as the percentage of seedlings that survived the 7-month experiment compared to the beginning of the experiment.

Each inoculum treatment reflects both fire severity and soil horizon, so we tested for differences in treatment means of seedling biomass (ten seedlings/inoculum type/host species) across a continuous fire-severity gradient (CBI) and soil horizon using regression and ANOVA, respectively. Initially, we explored models with site as a cofactor and found site not to be a significant factor. Because sites were chosen to represent different fire severities we did not include both site and CBI in the final model. All statistical analyses were performed in JMP 9.0.2 (SAS Institute Inc., 2010) with the exception of the multivariate analysis of fungal communities in PC-ORD 6.0.

## Results

**Fig. 2:**
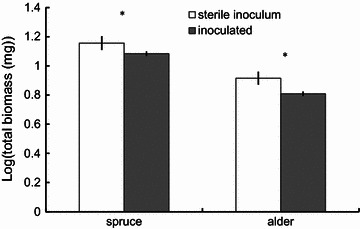
Inoculation reduces log-transformed seedling biomass (±1 SE) for both spruce and alder seedlings. *Asterisk* indicates significant differences (P < 0.01) between mean log-transformed biomass of seedlings inoculated and seedlings that received the sterile inoculum

Over 70 % of seedlings survived through the end of the experiment, and percent survivorship was not species-dependent (T = 1.50, P_(7, 6)_ = 0.184). Compared to controls inoculated with sterilized inoculum, inoculated soils from the ARF reduced log-transformed seedling biomass for both spruce and alder (full model F_(236, 2)_ = 88.7909, P < 0.000; inoculation F_(236, 1)_ = 5.642, P = 0.018; species F_(236, 1)_ = 169.752, P < 0.000) (Fig. 2).

### Treatment effects on seedling biomass

**Fig. 3:**
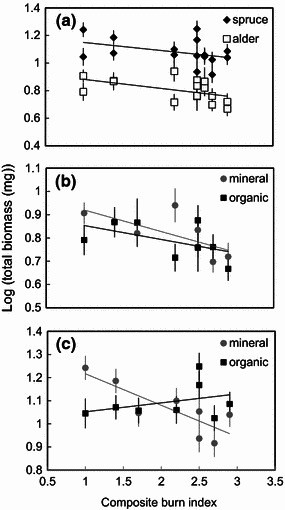
Effects of fire severity on log-transformed seedling biomass; **a** treatment means for spruce and alder biomass (±1 SE) decline with increasing fire severity; **b** alder biomass declines with increasing fire severity for seedlings grown with inoculum from mineral and organic soil horizons; **c** spruce biomass declines with increasing fire severity for seedlings grown with inoculum from the mineral soil horizon

The inoculation treatments given to the seedlings reflected both burn severity and soil horizon of a site. In general, fire severity had a stronger effect on seedling biomass than did soil horizon, particularly for alder. For inoculated alder we found a decrease in total seedling biomass with increased fire severity (F_(16, 1)_ = 4.976, P = 0.044) (Fig. 3a, b). Inoculated spruce log-transformed seedling biomass also decreased with increasing fire severity (F_(15, 1)_ =  4.175, P < 0.0636) but only for seedlings given mineral-soil inoculum (Fig. 3a, c). In contrast, soil horizon alone had no significant effect on inoculated log-transformed seedling biomass for either alder or spruce (alder F_(16, 1)_ = 0.728, P = 0.409; spruce F_(15, 1)_ =  0.704, P < 0.418). There was, however, a significant interaction between soil horizon and fire severity on inoculated spruce log-transformed seedling biomass (F_(15, 1)_ =  9.340, P < 0.010) (Fig. 3c).

### Fungal composition and effects on seedling biomass

In an exhaustive search for EM root tips, we failed to find any. However, we did observe numerous fungal hyphae and indications of some degree of fungal interaction with plant roots. Microscopic morphological examination (10–40×) of live, turgid root tips that had no root hairs from the root system of each spruce and alder seedling revealed no branched, swollen, or colored root tips with classic EM characteristics. Many of the spruce root tips were smooth with lighter coloration than lateral roots, while alder tips often had white cottony hyphae and dark areas of coloration, some of which appeared necrotic (Additional file 5). We further examined root tips on the compound scope (40–100×) to verify absence of root hairs and a fungal mantle and the presence of dark hyphae or necrosis. These morphological observations were supported by the molecular data. We successfully extracted DNA, amplified, and sequenced fungi from 199 seedlings and found no EM fungi associated with any seedling (Additional file 4). For roots that did not initially amplify, we performed multiple extractions and PCRs to ensure that seedlings indeed had no fungi associated with the root systems. We observed 115 fungal ribotypes across the two host species and obtained sequence identities for 28 ribotypes, including the most abundant ribotypes in our study. Although we were not able to match all ARISA ribotypes with sequence identities, the majority of dominant ARISA ribotypes were identified. Seventy-two percent of the total fluorescence from ARISA electropherograms was identified with matching sequences. The ribotypes that were matched with sequence IDs included a range of functional groups: seven of these are putative endophytes (including DSE), 13 are putative pathogens, two are saprotrophs, and six were identified to a deeper taxonomic level or were associated with multiple functional roles; none belonged to known EM taxa.

NMDS ordinations of all root tips sampled, revealed that fungal composition varied across seedlings and treatment. For inoculated spruce seedlings, fungal communities differed by fire-severity category (MRPP, A = 0.081, P = 0.009), but not soil horizon (MRPP, A = −0.012, P = 0.859). In contrast, for inoculated alder seedlings, there was significant variation in fungal composition by soil horizon (MRPP, A = 0.068, P = 0.015), but not for fire severity category (MRPP, A = −0.029, P = 0.779).

These results indicate variation in plant–fungal relationships depending on host species, but do not give an indication of the role fungal composition may promote or inhibit growth of seedlings post-fire. To determine the effect of fungal inoculum on log-transformed seedling biomass we used NMDS axes to represent differences in fungal composition across seedlings and treatments (Additional file 6). For future reference, we will refer to these axes as FC (fungal compositional) axes. Stepwise regression indicated that the model containing fungal composition represented by FC axes 1 and 2 generated the best-fit model, regardless of treatment. Log-transformed seedling biomass was significantly related to the fungal composition of inoculum for seedlings regardless of burn severity and soil horizon of a site (F_(114, 7)_ = 11.051, P < 0.000). We further investigated the effect of fungal composition for each treatment (fire severity and soil-horizon, Additional file 7a, b) on log-transformed seedling biomass for alder and spruce seedlings. For alder FC Axis 1 was the best fit, and was significantly related to biomass for alder seedlings (F_(16, 1)_ =  6.791, P < 0.021). For spruce the models containing FC axis 1, FC axis 2, or FC axis 3 provided an equal fit to spruce log-transformed seedling biomass, however none were significantly related to spruce log-transformed seedling biomass (F_(16, 1)_ =  0.506, P < 0.489). Overall, these results illustrate that fungal composition was significantly related to seedling biomass with treatment-level variations depending on seedling species. Alder biomass showed a strong relationship to the variation in fungal composition for individual treatments, while spruce seedling biomass did not.

**Fig. 4:**
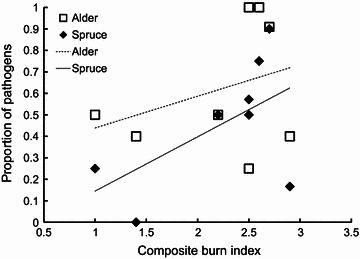
Proportion of identified ribotypes that are pathogenic vs. non-pathogenic fungi across the fire-severity gradient

The proportion of root-associated fungi identified as pathogens was directly related to fire severity across the gradient (Fig. 4, Total F_(8 1)_ =  4.9106, P < 0.0686, R^2^ = 0.45). Forty-one percent of the total fluorescence from ARISA profiles was attributable to fungal taxa, ribotypes, identified as pathogens. This relationship was stronger for fungi associated with spruce than those associated with alder seedlings (alder F_(8, 1)_ =  1.2742, P < 0.3021, R^2^ = 0.18; spruce F_(8, 1)_ =  6.7790, P < 0.0405, R^2^ = 0.53). Regardless of treatment, alder log-transformed seedling biomass was negatively correlated with the relative abundance of pathogens, (F_(89, 2)_ =  5.639, P < 0.020) and showed a marginally significant positive relationship to the relative abundance of the DSE functional group (F_(89, 2)_ =  3.025, P < 0.086). However, three DSE taxa were positively related to log-transformed seedling biomass: *Phialocephala fortinii* complex (ribotype 25, F_(89, 4)_ =  5.244, P < 0.025), *Cadophora finlandica* (ribotype 93, F_(89, 4)_ =  5.029, P < 0.024), and *Phialocephala* sp.(ribotype 90, F_(89, 4)_ =  15.743, P < 0.000). Relative abundance of these taxa did not vary significantly with fire severity. Together these results show that, as fire severity increases, fungal composition shifted to a greater proportion of known pathogens and log-transformed seedling biomass declined. Although some DSE taxa were positively correlated with seedling growth, pathogens had a strong negative influence on log-transformed seedling biomass.

## Discussion

Inoculation with burned soils reduced plant growth, apparently due to pathogenic effects of root-associated fungi. The proportional shift towards more pathogenic fungal symbionts with an increase in fire severity corresponded with reduced growth for alder and a weaker growth response of spruce seedlings inoculated with field soils. The significant colonization of seedlings by pathogens may relate to the ability of pathogens to disperse more widely and more quickly than many EM fungi [50, 51] and potentially DSE. Thirty-eight percent (5/13) of the sequenced fungi that we identified to at least the genus level as pathogens were also observed in soils along a trans-Arctic transect [26]. This suggests that the pathogens found in our study occur across the Arctic, and that pathogen propagules are robust, surviving for long periods in the soil, or distribute rapidly in tundra. The negative effect of pathogens on alder and spruce seedlings are consistent with the hypothesis of Blumenthal et al. [52] that stress-tolerating plant species are similarly susceptible to pathogens inside and outside of their native range, thus suppressing their capacity to invade novel environments.

Although the ARF post-fire soils were not an effective source of mutualist, EM fungi, inoculum for alder or spruce seedlings, they were a source of DSE inoculum. Of the DSE we identified in our bioassay, 100 % (11/11) were also observed in soils across the trans-Arctic transect [26]. DSE are widespread and can be more frequent at high-latitudes than classical mycorrhizal fungi [53]. Their proportional decline across the fire-severity gradient suggests that they are sensitive to fire disturbance, as we previously showed for EM fungi in post-fire treeline and tundra ecosystems [34, 41]. Colonization of roots by DSE is suggested to improve plant nutrition and biomass [13] and has been correlated with reduction of pathogenic root disease intensity [54]. Indeed, we found the relative abundance of three DSE taxa to be positively related to seedling biomass. We also observed an increase in the proportion of pathogenic fungi concurrent with a decline in DSE across the fire-severity gradient.

Because of the rarity of large-scale tundra fire disturbances for at least 11,000 years [22], mutualist soil biota inoculum in Arctic Alaska may lack fire-specialist taxa that are either resilient to fire or are stimulated by fire. The site of the ARF had not burned for the preceding 5000 years [23], in stark contrast to the average fire return interval of 150 years in the boreal forest [55]. Our previous field research suggests that surviving mycorrhizal shrubs are a more likely source of EM inoculum than are spores or sclerotia from the post-fire RPC [31] in soils following tundra fires [34, 41]. Mycelial inoculum sources may be limited in tundra because of low density of EM host plants in many tundra ecosystems. These shrubs can take up to a decade to return to pre-fire densities after wildfire [56]. The effectiveness of the RPC as an inoculum source after fire is often restricted by the availability of spores and sclerotia [57] and by the efficacy of these inoculum sources with certain host plants [58]. Instead of a robust EM RPC community providing beneficial inoculum, we observed DSE colonization in our bioassay, suggesting that other potentially beneficial mycobionts, DSE, may be less sensitive to fire or that they disperse more widely and/or quickly than EM fungi.

Although the lack of mycorrhizal development in our study could also have been an artifact of the growth chamber conditions, other studies have shown that, consistent with our methods, the intermittent application of soluble fertilizer (Castellano et al. 1985) and the storage of soil inoculum (Nunez et al. 2009) or spore slurries (Castellano et al. 1985) for comparable or longer time periods did not inhibit the formation of mycorrhizas on small, first-year seedlings. We conclude that these patterns are ecologically relevant for three main reasons: (1) in this study both biomass and fungal composition were related to fire severity; (2) in companion field studies we observed fire-severity effects on inoculum composition for naturally established tundra shrubs and treeline shrubs and seedlings [34, 41] and treeline seedling biomass was correlated with post-fire fungal composition [41]; and (3) our inoculation and fertilization methods were consistent with other studies where EM formation was observed.

**Fig. 5:**
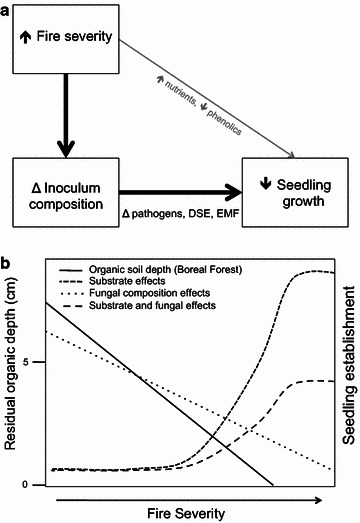
Conceptual model of fire-severity effects on vegetation change in the Arctic can be assessed through **a** factors influencing seedling growth and **b** the net outcome of substrate and microbial community factors on seedling establishment in non-shrubby tundra. **a** The *thickness of the arrows* indicates strength of the causal pathway. *Grey text* indicates alternative mechanisms influencing seedling biomass after fire, whereas *black text* indicates the causal pathway investigated and supported in this study. **b** Substrate effects refer to the positive effect of burn severity on exposure of high-quality, mineral-soil seedbeds

We suggest that the effect of fire severity on seedling biomass in a controlled bioassay was due to fire-severity effects on fungi not variability in available nutrients or inhibitory phenolics in the soil across the fire-severity gradient (Fig. 5a). We dismiss the idea of a nutrient effect because the amount of field soil provided for inoculation was small (12 ml), and all seedlings in the experiment were fertilized. Similarly, phenolic-effects are not likely in this ecosystem because fire consumption of the organic horizon volatilizes inhibitory allelochemicals and phenolics in litter and soil [25], and charcoal sorbs inhibitory compounds [59]. This suggests that a fire effect on fungal community composition is the most likely explanation for the fire-severity effect on seedling biomass. Along these lines, we observed reduced seedling biomass and changes in the proportion of functional groups of root-associated fungi as fire severity increased across the gradient of sites. In addition, seedling biomass was related to fungal composition. Hence, we believe that decreased biomass along the fire-severity gradient is attributable to changes in fungal composition related to fire severity.

The influence of fire severity, however, differed between the two host species. Alder seedling biomass declined as expected with increasing fire severity as the proportion of fungal pathogens increased. There was a strong relationship between fungal composition and mean biomass for each treatment, which appears to drive the effect of fire severity on alder seedling biomass. Spruce seedlings, however, responded to fire severity in a more complex way. Spruce biomass declined with increasing fire severity, though to a lesser degree than alder, with mineral-soil inoculum. In contrast to alder, the mean biomass of spruce seedlings did not significantly correlate to fungal composition, even though spruce seedling biomass declined with increasing fire severity of the inoculum and variability in fungal inoculum differed by fire-severity category. Two potential explanations are that (1) spruce-seedling biomass was reduced by an unidentified landscape factor affecting soil inoculum that was highly correlated with fire severity; or (2) particular fungal taxa instead of overall fungal composition exerted a strong effect on spruce growth. However, analysis of the ribotypes that were matched with sequences did not reveal that the relative abundance of a specific taxon had a strong effect on biomass of spruce seedlings inoculated with organic soils.

Overall, our findings illustrate that in a highly controlled growth chamber setting seedling growth was reduced when grown in soils from sites with increasing tundra fire severity, likely through growth reduction by root-associated fungal pathogens or fungi with pathogenic effects. Although there is uncertainty with translating growth chamber experiments to outcomes in the field, our results suggest that the expected positive effects of fire severity on tree and shrub establishment in tundra after fire [20, 60] may be dampened by changes in soil biota associated with fire severity. Field studies are necessary to determine whether these negative effects of post-fire fungi on seedling growth occur in a complex post-fire field environment, outweigh the potential positive effects due to pathogen release or the benefits of mutualist fungi resilient to tundra fires, and/or constrain seedling establishment beyond the native range in non-shrubby tundra. In a field study in the boreal forest, Johnstone & Chapin [60] observed decreased tree seedling establishment at extremely high-severity sites, despite nearby EM nurse plants that were potential sources of EM inoculum. This observed negative effect of high fire severity on seedling recruitment in the field might reflect a shift in mycobiont composition such as we observed that reduces seedling growth (Fig. 5b).

Understanding the influence of fungi on successful seedling establishment is important to forecasts of spruce migration and alder expansion in Arctic Alaska and associated ecosystem feedbacks. Both spruce migration and alder expansion have large ecosystem impacts due to changes in carbon storage, albedo, ecosystem services, and nutrient cycling. In particular, the expansion of alder has a significant influence on nitrogen and phosphorus cycling because of its role as a nitrogen fixer. On a global scale, Harsch et al. [2] found that 2 of 166 treeline sites receded since 1900 AD and that both of these sites showed evidence of disturbance. These authors infer that disturbance legacies do not likely affect the probability of advance, and instead influence initial recruitment and lag times between warming and treeline advance. Currently 2.3 % of tundra has been converted to forest in Alaska [61]. To extrapolate from our study, we would expect the rate of tree migration and shrub expansion to be constrained by pathogenic effects of soil biota and mutualist limitation after high-severity fires. However, if boreal EM fungi co-migrate or EM tundra shrubs provide surrogate sources of inoculum on the landscape under low and moderate severity fires [34], we would expect that boreal forest mycobionts may then facilitate vegetation change at and beyond current treeline, reducing lag times by facilitating initial recruitment. Thus, mutualistic and pathogenic symbionts may either constrain or facilitate vegetation change depending on fire severity or other contexts.

## Conclusions

This study provides an initial assessment of post-fire plant–fungal interactions in a controlled growth chamber setting for two plant species expected to expand into tundra with future scenarios of warming and wildfire. We found that seedling biomass was related to the composition of root-associated fungi and that fungal composition in our bioassay inoculum shifted across the fire-severity gradient. Decreased seedling biomass across the fire-severity gradient suggests that fire-severity effects on plant–fungal interactions may dampen seedling performance and thus establishment success. However, follow-up field studies are necessary to evaluate the relative importance of the negative effects of plant–fungal interactions on seedling performance within different post-fire contexts.

## Availability of supporting data

The datasets supporting the results of this article are available in the following repositories:

Sequences for each OTU have been archived with GenBank under accession numbers KF660543–KF660580.

Binned ARISA ribotype abundance data have been archived with the Bonanza Creek LTER and the LTER Network Data Portal doi:10.6073/pasta/a131c0d6707b6aa746dfe0265141ad43.

Environmental and fire-severity data are published in Jandt et al. 2012 and available through www.Frames.gov.

## Additional files

10.1186/s12898-016-0075-y Site descriptions for eight sampling sites grouped by fire-severity categories within the Anaktuvuk River Fire burn scar. We classified vegetation communities using the Viereck (1992) vegetation classification: 2C2A = open low scrub mixed shrub-sedge tussock tundra, 2C2H = open low scrub willow-sedge shrub tundra, 3A21 = mesic graminoid herbaceous sedge-birch tundra, 3A2D = mesic graminoid herbaceous tussock tundra, and 3A3 = wet graminoid herbaceous tundra. Fire-severity categories were assigned based on composite burn index and field descriptions.
10.1186/s12898-016-0075-y Detailed methods of molecular techniques used to characterize fungal communities.
10.1186/s12898-016-0075-y Biological key showing ribotype ID, abundance, range of fungal ITS fragment lengths, associated OTU identity, and the best match description as reported in GenBank.
10.1186/s12898-016-0075-y Operational Taxonomic Units at 97 % sequence similarity for fungi associated with seedlings inoculated with soils from the Anaktuvuk River Fire.
10.1186/s12898-016-0075-y Photographs of typical morphologies of seedling root systems: a-c alder; d-f spruce.
10.1186/s12898-016-0075-y Description of final solutions for nonmetric multidimensional scaling ordinations of fungal composition for all seedlings and treatment x species combinations.
10.1186/s12898-016-0075-y Biplots of nonmetric multidimensional scaling ordinations of fungal communities associated with a). alder and b). spruce seedlings inoculated with soils from the Anaktuvuk River Fire and site characteristics. Active layer = depth of the unfrozen soil at time of sampling; dNBR = differenced normalized burn ratio of the sampling site; CBI = composite burn index of the sampling site.

## Abbreviations

ARISA: automated ribosomal intergenic spacer analysis
ARF: Anaktuvuk River fire
CBI: composite burn index
DSE: dark septate endophytes
EM: ectomycorrhizal
ERM: ericoid mycorrhizal
ITS: internal transcribed spacer
MRPP: multiple-response permutation procedures
NMDS: nonmetric multidimensional scaling
RPC: resistant propagule community
