# Supplementary material for: Fire-severity effects on plant–fungal interactions after a novel tundra wildfire disturbance: implications for arctic shrub and tree migration
Source: BMC Ecol. 2016 May 11;16:25. doi: 10.1186/s12898-016-0075-y (PMC4865011; doi:10.1186/s12898-016-0075-y)
Supplement: Supplementary file 2 — 10.1186/s12898-016-0075-y Detailed methods of molecular techniques used to characterize fungal communities. [file 12898_2016_75_MOESM2_ESM.docx]

**Additional file 2:** *Detailed methods of molecular techniques used to characterize fungal communities.*

DNA extraction

Root tips were suspended in 250 μl lysis buffer and ground in a 0.6 ml microfuge tube with a Kontes pellet pestle (Kimble Chase, Vineland, NJ, USA). We extracted DNA using the Qiagen DNEasy Plant Mini Kit (QIAGEN Inc., Valencia, California, USA) according to the manufacturer’s instructions. DNA extraction was repeated 2-3 times for samples that showed no ARISA peaks to confirm that no fungal DNA was present.

ARISA

Twenty-five μl PCR reactions were prepared containing 0.65 mM MgCl_2_, 0.2 mM dNTPs, 0.05 μM forward primer FAM-ITS1F (CTTGGTCATTTAGAGGAAGTAA [1] labeled on the 5’ end with FAM, a fluorescein amidit (Applied Biosystems, Carlsbad, CA)), 0.05 μM reverse primer ITS4 (TCCTCCGCTTATTGATATGC [2], 0.06 mg/ml bovine serum albumin, 0.15 μl JumpStart RED Taq (Sigma-Aldrich, St. Louis, MO, USA), 1X JumpStart RED Taq buffer and 5 μl of DNA extract. Reactions were prepared in 0.2 μl tubes and ran in an MJ Research PTC-225 thermal cycler as follows: 96°C for 3 min, 35 cycles of 94°C for 30s, 52°C for 30s, then 72°C for 3 min, followed by 72°C for 10 min [3]. We diluted the post-PCR DNA extracts (1/10) in ultrapure water based on our own optimization procedure for adjusting PCR product to concentrations appropriate for comparison with the size standard used for ARISA. One microliter of each sample was then mixed with 14.25 µl of formamide and 0.75 µl of size standard (MapMarker 1000 X-rhodamine, BioVentures, Murfreesboro, TN, USA). These reactions were heated to 95ºC for 5 minutes, then immediately placed on ice until run through an ABI 3100 Genetic Analyzer (Applied Biosystems, Foster City, NJ, USA; Pop6, 50cm array, T-RFLP_1500 protocol). PCR reactions were repeated 2-3 times for samples that showed no ARISA peaks to confirm that no fungal DNA was present.

ITS sequencing

To assign taxonomic identities to the dominant ARISA fragments, fungal ITS gene region sequences were obtained by PCR amplification, as described earlier, except that the PCR primers did not have a fluorescent moiety added to the forward primer. Fungal ITS sequences were obtained directly from pooled root samples in cases where only one dominant ARISA peak was seen. PCR products were shipped on wet ice overnight for sequencing at Functional Biosciences Inc. (Madison, WI, USA).

Bioinformatics

Sequences were assembled in Codoncode Aligner 3.7 (CodonCode Corporation, Dedham, MA, USA) using PHRAP. We used in-house perl scripts to mask low-quality bases based on phred scores (cutoff Q20), orient, and purge sequences containing >3% Ns after end-trimming [4]. We grouped sequences into Operational Taxonomic Units (OTUs) using CAP3 [5] at 97% sequence similarity. A representative sequence was selected for each OTU after manual inspection in SeAl alignment software [6]. To assign taxonomic identities we compared the representative sequence for each OTU to ITS sequences from GenBank, utilizing a curated specimen fungal ITS search filter (http://www.borealfungi.uaf.edu/). The top 10 hits from the BLAST search were assessed for the coverage between the query and the hit sequences and the % identity. When the top 10 hits did not have high coverage, % identity, or consistency in identification, we built maximum likelihood trees to resolve the identity of our queried sequence using the top vouchered and isolate sequences from the curated database on the Fungal Metagenomics Project website (http://www.borealfungi.uaf.edu/). To construct trees, we aligned sequences in MUSCLE [7] and used the maximum likelihood method with default settings (GTR+G+I) in Garli v.1.0 [8]. We manipulated the tree, including midpoint rooting, in FigTree v1.3.1 [9]. Nomenclature for each OTU follows Timling *et al* [10]. Sequences for each OTU have been archived with GenBank under accession numbers listed in (see Table S3). Sequence lengths were computed using the DNA stats function under the sequence analysis menu at DNA 2.0 Bioinformatics toolbox (https://www.dna20.com/index.php?pageID=216). Direct sequences were matched up with the dominant ribotype in the ARISA profile based on fragment length.

We used GeneMapper 3.7 (Applied Biosystems, Foster City, NJ, USA) to read each ARISA electropherogram. In our study, ARISA was used to estimate community composition by determining the fragment length heterogeneity of the nuclear ribosomal ITS region for fungi present in the pooled community sample. The peak heights for each fragment were relativized by dividing the fluorescence height for each peak by the total fluorescence height for a sample profile [11]. Fragment length indicates the identity of a fungal taxon (henceforth ‘ribotype’) and peak height gives a measure of the relative abundance of that taxon. Raw fragment sizes and associated peak heights were exported from GeneMapper. We used raw ARISA ribotypes lengths and analysis of corresponding OTU sequence lengths to bin fragment reads into surrogates for fungal “species” (see Table S3). In general, ARISA ribotypes were binned at 1 bp bin size where fragment size was rounded to the nearest integer. Fragment detections within 0.5 bp are accepted to belong to the same ribotype, because the machine error in size estimation is below 0.5 bp. This is often the default binning threshold in ARISA and TRFLP software [12]. However, we found a few exceptions to the 1bp bin width rule where biological variation in sequence length within an OTU was greater than 1 bp. When OTU sequence length variation indicated greater than 1 bp size variation for an OTU with 97% similarity of sequence identity we expanded the 1 bp ribotype bin to include ribotypes known to belong to the same OTU. Binned ribotype abundance data have been archived with the Bonanza Creek LTER <http://www.lter.uaf.edu/data_b.cfm> and were used in multivariate analysis of fungal communities.

**References**

1. Gardes M, Bruns TD: **ITS primers with enhanced specificity for basidiomycetes - application to the identification of mycorrhizae and rusts** *Mol Ecol* 1993, **2**(2):113-118.

2. White TJ, Bruns T, Lee S, Taylor JW: **Amplification and direct sequencing of fungal ribosomal RNA genes for phylogenetics**. In: *PCR Protocols: A Guide to Methods and Applications.* Edited by Innis MA, Gelfand DH, Sninsky JJ, White TJ. New York: Academic Press; 1990: 315-322.

3. Bent E, Taylor DL: **Direct amplification of DNA from fresh and preserved ectomycorrhizal root tips**. *Journal of Microbiological Methods* 2010, **80**(2):206-208.

4. Taylor DL, Houston S: **A Bioinformatics Pipeline for Sequence-Based Analyses of Fungal Biodiversity**. In: *Fungal Genomics.* Edited by Xu J-R, Bluhm BH, vol. 722. New York, New York, USA: Humana Press; 2011: 141-155.

5. Huang X, Madan A: **CAP3: A DNA Sequence Assembly Program**. *Genome Research* 1999, **9**:898-877.

6. **Sequence Alignment Editor.** <http://tree.bio.ed.ac.uk/software/seal/> [<http://tree.bio.ed.ac.uk/software/seal/>]

7. Edgar RC: **MUSCLE: multiple sequence alignment with high accuracy and high throughput**. *Nucleic Acids Research* 2004, **32**(5):1792-1797.

8. Zwickl DJ: **Genetic algorithm approaches for the phylogenetic analysis of large biological sequence datasets under the maximum likelihood criterion**. Austin: The University of Texas at Austin; 2006.

9. Rambaut A: **FigTree: Phylogenetic tree production.** <http://tree.bio.ed.ac.uk/software/figtree/>. 2009.

10. Timling I, Dahlberg A, Walker DA, Gardes M, Charcosset JY, Welker JM, Taylor DL: **Distribution and drivers of ectomycorrhizal fungal communities across the North American Arctic**. *Ecosphere* 2012, **3**(11):3:art111. <http://dx.doi.org/110.1890/ES1812-00217.00211>.

11. Fisher MM, Triplett EW: **Automated approach for ribosomal intergenic spacer analysis of microbial diversity and its application to freshwater bacterial communities**. *Applied and Environmental Microbiology* 1999, **65**(10):4630-4636.

12. Dunbar J, Ticknor LO, Kuske CR: **Phylogenetic specificity and reproducibility and new method for analysis of terminal restriction fragment profiles of 16S rRNA genes from bacterial communities**. *Applied and Environmental Microbiology* 2001, **67**(1):190-197.
