# Supplementary figures and images for: Fire-severity effects on plant–fungal interactions after a novel tundra wildfire disturbance: implications for arctic shrub and tree migration
Source: BMC Ecol. 2016 May 11;16:25. doi: 10.1186/s12898-016-0075-y (PMC4865011; doi:10.1186/s12898-016-0075-y)

(a)

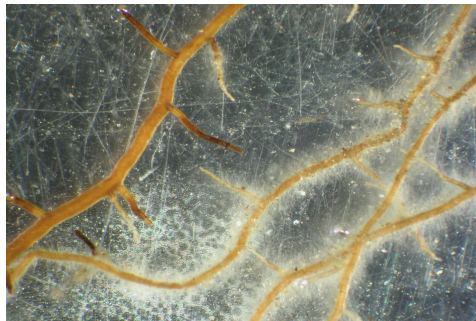

(d)

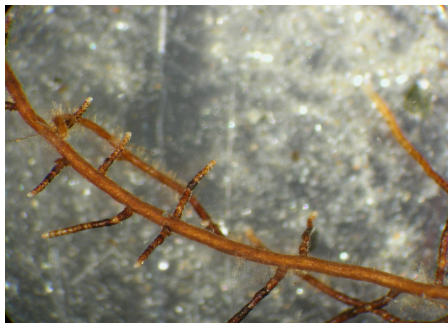

(b)

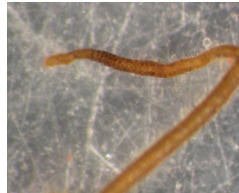

(c)

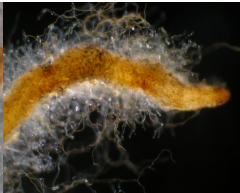

(e)

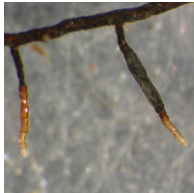

(f)

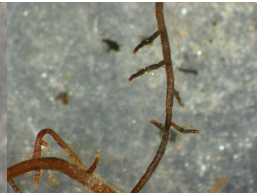

Supplement: Supplementary file 5 — 10.1186/s12898-016-0075-y Photographs of typical morphologies of seedling root systems: a-c alder; d-f spruce. [file 12898_2016_75_MOESM5_ESM.pdf]

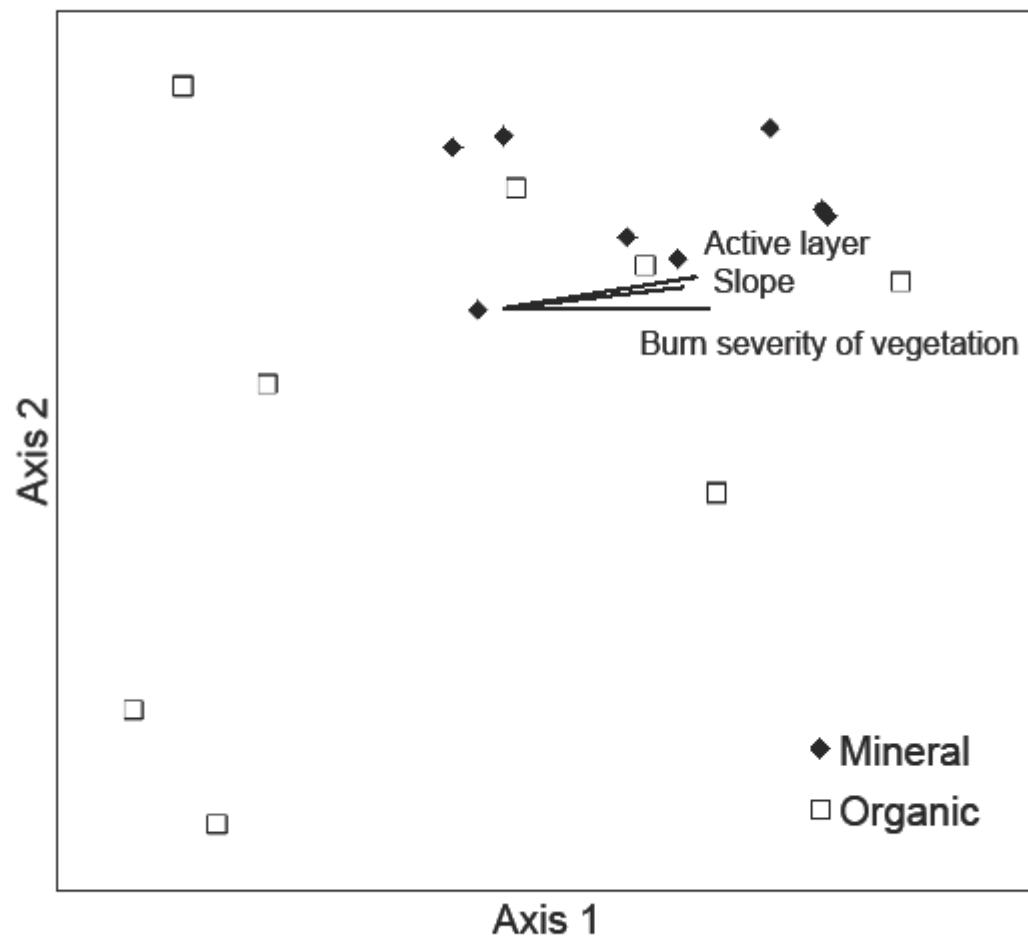

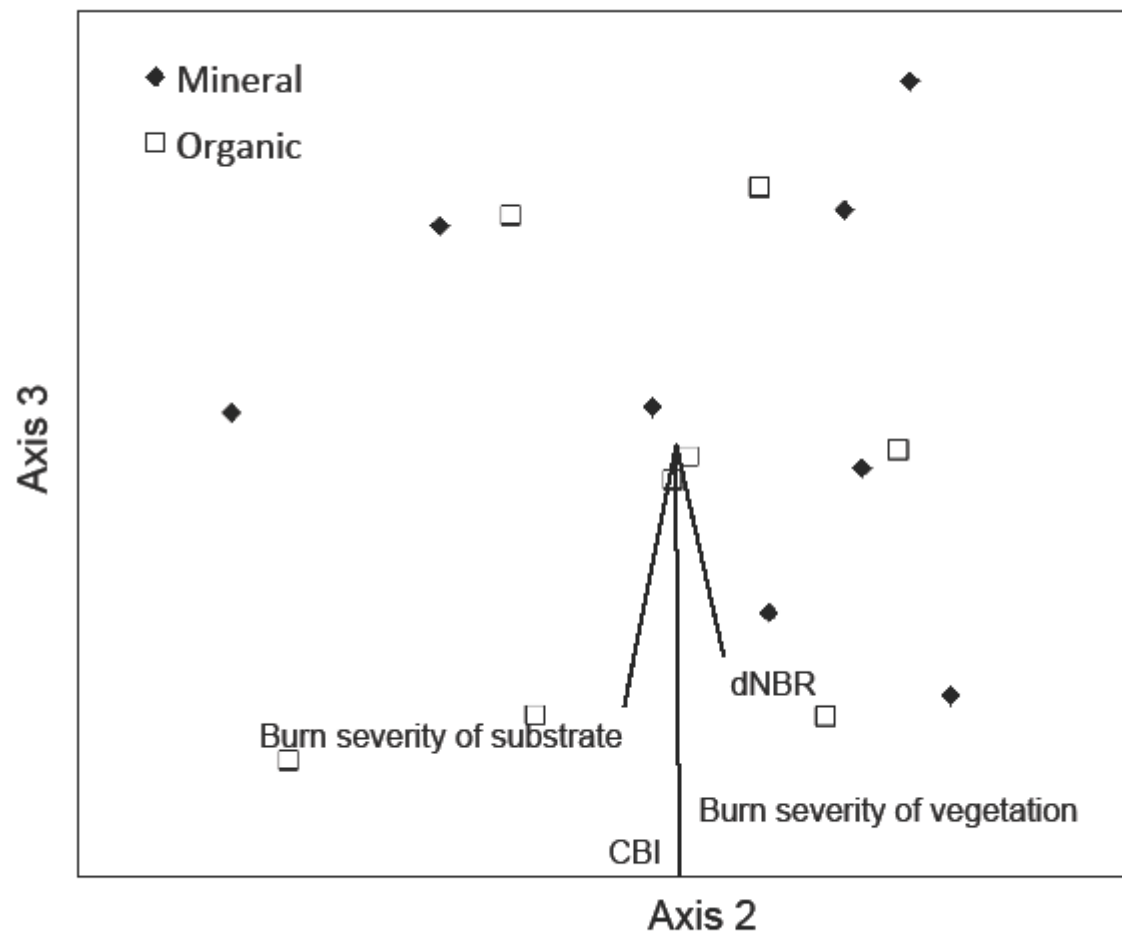

Supplement: Supplementary file 7 — 10.1186/s12898-016-0075-y Biplots of nonmetric multidimensional scaling ordinations of fungal communities associated with a). alder and b). spruce seedlings inoculated with soils from the Anaktuvuk River Fire and site characteristics. Active layer = depth of the unfrozen soil at time of sampling; dNBR = differenced normalized burn ratio of the sampling site; CBI = composite burn index of the sampling site. [file 12898_2016_75_MOESM7_ESM.pdf]
